# Collective Motion of Swarming Agents Evolving on a Sphere Manifold: A Fundamental Framework and Characterization

**DOI:** 10.1038/srep13603

**Published:** 2015-09-09

**Authors:** Wei Li

**Affiliations:** 1 Department of Control and Systems Engineering, Nanjing University, China.

## Abstract

Collective motion of self-propelled agents has attracted much attention in vast disciplines. However, almost all investigations focus on such agents evolving in the Euclidean space, with rare concern of swarms on non-Euclidean manifolds. Here we present a novel and fundamental framework for agents evolving on a sphere manifold, with which a variety of concrete cooperative-rules of agents can be designed separately and integrated easily into the framework, which may perhaps pave a way for considering general *spherical collective motion* (SCM) of a swarm. As an example, one concrete cooperative-rule, i.e., the *spherical direction-alignment* (SDA), is provided, which corresponds to the usual and popular direction-alignment rule in the Euclidean space. The SCM of the agents with the SDA has many unique statistical properties and phase-transitions that are unexpected in the counterpart models evolving in the Euclidean space, which unveils that the topology of the sphere has an important impact on swarming emergence.

Collective motion (CM) of self-propelled agents is an important and fascinating emergent phenomenon in nature and artificial world, and has attracted increasing interests in the past two decades in many disciplines, e.g., physics, mathematics, biology, and robotics^1^,^2^,^3^,^4^,^5^,^6^,^7^,^8^,^9^,^10^,^11^,^12^,^13^,^14^,^15^,^16^,^17^,^18^,^19^,^20^,^21^,^22^,^23^,^24^,^25^,^26^,^27^,^28^,^29^,^30^,^31^,^32^,^33^,^34^,^35^,^36^,^37^,^38^,^39^,^40^,^41^,^42^,^43^,^44^,^45^,^46^,^47^,^48^. However, almost all work considers CM of agents evolving in either the one-dimensional^19^, two-dimensional (most references), or three-dimensional^8^,^14^,^27^,^28^ (for abbreviation, 1D, 2D, 3D, respectively) Euclidean space, in either a discrete^8^,^14^,^21^,^22^,^23^,^24^,^25^,^26^,^27^,^28^,^34^,^35^,^36^,^48^, or continuous^9^,^15^,^37^,^38^,^39^,^40^,^44^,^45^ or continuum^46^,^47^ formulation. Here *the meaning of the discrete, continuous, and continuum formulations* is that, the kinematics/dynamics of agents is described by discrete difference equations (DDEs), ordinary differential equations (ODEs), and partially differential equations (PDEs), respectively, typically in the Euclidean space. Some work also considers agents evolving in a *discrete topological space* (e.g., agents move on the vertices of a lattice^16^), which is a non-continuum space. There is rare concern on swarming agents that evolve on a non-Euclidean manifold^29^, especially from the perspective of statistical physics. For convenience, refer CM in the Euclidean space as ECM.

CM of agents on a sphere (an important non-Euclidean manifold), which itself is an interesting topic, has important implications in analyzing many types of self-propelled agents or continuum-flows (e.g., possibly by a coarse-grained approximation^45^) evolving on a sphere. For example, fluid patterns and evolution predictions of the atmosphere on the Saturn planet, the current evolution and patterns on the surface of a soap bauble or a water ball in the outer space (which is a water sphere without the effect of the gravity), and even CM of unmanned aircraft near the surface of a planet.

Compared with the ECM, formulation and characterization of the *spherical collective motion* (SCM) are much different and complex, since the physical topologies of the sphere and the Euclidean space are distinct. The configuration-state (i.e., position and moving direction) space of an agent on a sphere is the tangent bundle of the sphere. Furthermore, formulation of cooperation will further induce much complexity.

The main contributions of this paper are in the following two aspects.

1. In this paper, we are first interested in modeling a simplest possible yet effective framework for the SCM of multiple agents driven by a *generic cooperative-rule* (GCR), in which a set of the framework-rules describes spherical motion of each agent evolving on its own great-circle at each step, with a structure for integration of the GCR into the framework. As a result, the framework is *versatile* in the sense that: any instance of the GCR (or called a concrete cooperative-rule) can be then designed separately and integrated easily within the framework. Moreover, design of a concrete cooperative-rule for the SCM is rather similar (to a certain degree) as for the case of agents in the Euclidean space, which will add further convenience. New notions, phenomena, and characterizations of the SCM are then provided that are distinct from the cases of the ECM.
2. As an important example of the GCR, the *spherical direction-alignment* (SDA) is then provided, which corresponds to the Euclidean direction-alignment (EDA)^22^ (that was first presented by Vicsek etc., and has been widely adopted in the past decades in many disciplines) of agents that is fundamental for the ECM. The SCM with the SDA has many unique characteristics that are unexpectedly distinct from the counterpart ECM model with the EDA, which unveil that the topology of the sphere has an important impact on swarming emergence.

## Framework

### Definitions and Notations

Without loss of generality, assume the sphere is located at the origin of the 3D Euclidean space with radius *r* > 0. Define 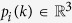 as the position of agent *i*, *i* = 1, 2, …, *n*, on the sphere at step *k* in the Cartesian coordinates, 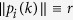 for all *i*, *k*, *k* = 0, 1, 2, …, where 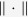 is the Euclidean norm,


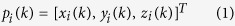


and 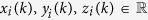 are the X-Y-Z coordinates of position *p*_*i*_(*k*) in the Cartesian coordinates, respectively. Notice that notation *p*_*i*_(*k*) also represents the position vector that starting from the center of the sphere to the position of agent *i*, according to the context. Denote *T*_*i*_(*k*) as the tangent plane to the sphere at position *p*_*i*_(*k*).

We use the notion “*direction*” to express the heading-orientation of an agent in the 3D Euclidean space, which corresponds to the heading angle that is valid in the 2D Euclidean space, and the 3D direction is a necessity to avoid possible *symmetry-breaking* control laws^28^. The *direction* of agent *i* at step *k*, denoted as 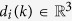, implies that:

1. it is a vector with a *unitary* magnitude: 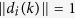; and
2. it is constrained on the tangent plane *T*_*i*_(*k*), i.e., it is perpendicular to the position of this agent: *d*_*i*_(*k*)  ┴  *p*_*i*_(*k*).

The *adaptive velocity* of agent *i* has two meanings:

1. an adaptive direction according to a certain rule; and
2. an adaptive speed (here speed means the magnitude of velocity) *v*_*i*_(*k*), typically measured by the step-size at each step in a discrete formulation.

For spherical motion of an agent, there are two required configuration constraints: the position-constraint 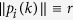, and the direction-constraint *d*_*i*_(*k*) ┴ *p*_*i*_(*k*).

### Framework

The framework, as one of the main focuses in this paper, is described as the iterative equations as follows.

First, for clarity and conciseness, define the vectorial function 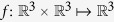 as:


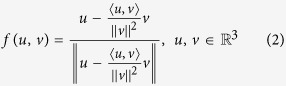


where the notation 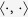 means the inner product in the 3D Euclidean space, which has the *physical meaning* that: it calculates the component of vector *u* that is perpendicular to vector *v*, and then makes this component normalized via the division of the magnitude 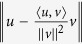 of the component, provided that *u*, *v* are not parallel (i.e., 
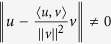
).

Consider the *framework-rules* of the SCM of agents in a discrete formulation, the initial positions *p*_*i*_(0), directions *d*_*i*_(0), and speeds *v*_*i*_(0) of all agents are given for the start of the iteration. Without loss of generality, assume *v*_*i*_(0) = 0 for all *i* and the time interval Δ*t* is a unit, i.e., Δ*t* = 1. The next position *p*_*i*_(*k* + 1), direction *d*_*i*_(*k* + 1), and speed *v*_*i*_(*k* + 1) of every agent *i* at step *k* + 1 that starting from position *p*_*i*_(*k*) with direction *d*_*i*_(*k*) and speed *v*_*i*_(*k*), are described by


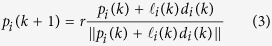



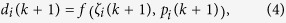



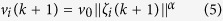



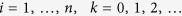
 where in (3) notation 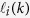 is called the *mapped-Euclidean-step-size* that is defined as


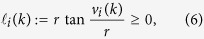


which ensures the *spherical-step-size* of agent *i* to be the speed *v*_*i*_(*k*), i.e., the *great-circle-distance* (GCD) between two steps *p*_*i*_(*k*) and *p*_*i*_(*k* + 1) is just *v*_*i*_(*k*)Δ*t* = *v*_*i*_(*k*); *v*_0_ is a constant (*v*_0_ ≪ *r*) that represents the maximum-possible speed of all agents; the exponent *α* ≥ 0 characterizes the adaptivity of speed of agents.

### Generic Cooperative-Rule and Notion of Spherical Approaching-Direction

In the framework, 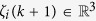 represents the GCR of agent *i* at step *k* + 1:


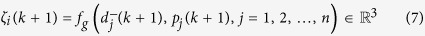


where 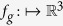 is a generic function to be designed for a certain expectation of the SCM, which may probably use the notion *spherical approaching-direction* 
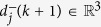
:


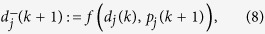


it is the very direction of agent *j* when this agent just reaches at next position *p*_*j*_(*k* + 1), while before it switches to its new direction *d*_*j*_ (*k* + 1) at that position (the superscript “^−^” in 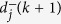 means “the left limit” at that position, hence the word “approaching” is used in this notion), the invariant quantity is


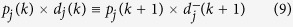


where notation × means the cross product of two vectors. As a comparison, the notion “approaching-direction” is not needed in the Euclidean space, since any agent moves in a straight line to its next position without direction change during the traveling of any adjacent steps, thus 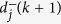 just reduces to be the starting direction *d*_*j*_(*k*) at position *p*_*j*_(*k*) in the Euclidean space.

In design of *ζ*_*i*_(*k* + 1), generally 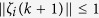 is required; and *ζ*_*i*_(*k* + 1) ┴ *p*_*i*_(*k* + 1) is preferred, but not necessarily required, owning to the perpendicular ramification by rule (4) using the function *f*.

In the SCM framework and the GCR, note that 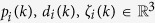 are the 3D vectorial variables; *f*, *f*_*g*_ are two vectorial functions; 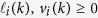 are the scalar variables; while *r*, *α*, *v*_0_ > 0 are the scalar constants.

### Interpretations

First, every agent *i* in the swarm with rules (3)(4) and (6) is constraint to move on the sphere along the moving direction *d*_*i*_(*k*) [that starts at position *p*_*i*_(*k*) at step *k* with the speed (or the spherical-step-size) *v*_*i*_(*k*)] from position *p*_*i*_(*k*) to position *p*_*i*_(*k* + 1); and then it will move along the moving direction *d*_*i*_(*k* + 1) [that starts at position *p*_*i*_(*k* + 1) at step *k* + 1 with the speed *v*_*i*_(*k* + 1)] to the next position, and so on. The formulation is robust in the sense that, any uncertain or noise in the system (although which may possibly influence the behaviors of the SCM of the agents) will not drive the agents away from the sphere.

The direction *d*_*i*_(*k* + 1) of agent *i* is determined by the cooperative-rule *ζ*_*i*_(*k* + 1) and then calculated with the unitary and perpendicular ramification by rule (4).

The physical meaning of *ζ*_*i*_(*k* + 1) can be viewed as a certain form of the local polarization surrounding agent *i* (according to a concrete form of function *f*_*g*_) when all agents are just *approaching* to their next positions at step *k* + 1 (refer to the notion *spherical approaching-direction*). The vectorial information of *ζ*_*i*_(*k* + 1) is used to determine direction *d*_*i*_(*k* + 1) of agent *i* according to rule (4), its magnitude 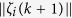 determines *v*_*i*_(*k* + 1) by rule (5). The physical meaning of exponent *α* > 0 implies that, when *ζ*_*i*_(*k* + 1) shows a strong polarization, i.e., 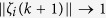, agent *i* naturally moves faster; while *ζ*_*i*_(*k* + 1) shows no or a weak polarization, i.e., 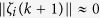, agent *i* naturally hesitates and is confused to move at such singularity situation, thus *v*_*i*_(*k* + 1) ≈ 0; this phenomenon is called the adaptive velocity mechanism (AVM)^27^,^28^.

It is easy to express a constant-speed-motion of agents, which is the usual assumption in literature (the advantage of the AVM vs the constant-speed-motion is illustrated in^27^). That is, let


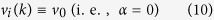


for all *i*, *k* that replacing the rule (5), thus the equations (3) (4) describe the agents with constant speed *v*_0_ on the sphere, with the rule (6) for different *i*, *k* reduces to be a constant:


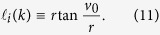


The framework is versatile in that, a variety of concrete cooperative-rules of *ζ*_*i*_(*k* + 1) can be designed separately and integrated easily into the framework.

Finally, a sphere manifold is a closed set, thus the periodic boundary condition (e.g., in^22^ and many other papers) for agents in the Euclidean space is not needed when considering SCM. Certainly, one may apply the periodic boundary condition or other boundary conditions to a designated region of interest on the sphere and investigate the agents evolving on that region instead of the whole sphere, this is out of the scope of this paper.

## Formulation of the SDA

As an important example of the GCR, consider


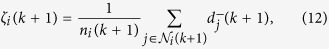


where

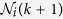
 is the neighbour set of agent *i* at step *k* + 1, and 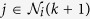 if the GCD between agents *i*, *j* is less than threshold *r*_0_, where 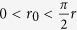,
- *n*_*i*_(*k* + 1) is the number of agents (including agent *i*) in the neighboring set 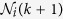, thus *n*_*i*_(*k* + 1) ≥ 1.

Note that the rule (12) calculates the average of the *spherical approaching-directions* (8) of agents in 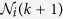. 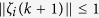, so 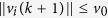.

For convenience, the rule (12), together with the unitary and perpendicular ramification (4), is called the SDA (the noise will be discussed in the following). Its physical meaning is that: *every agent adopts the very direction at each step, which is derived by averaging the spherical approaching-directions of its neighboring agents at that step, then with the unitary and perpendicular ramification*.

Note that the SDA rule (12) is equal to the expression:


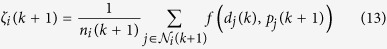


using the function *f*.

As a comparison, recall the fundamental EDA in the Euclidean space, with its physical meaning as that: *every agent adopts the average direction of its neighboring agents at each step* (that was first presented by Vicsek etc. in^22^, and widely adopted in the past decades in many disciplines).

### SDA With Noise

Noise is a common factor in CM of agents. Expression of noise in the SDA is also complex, compared with the EDA in the Euclidean space.

**Figure 1:**
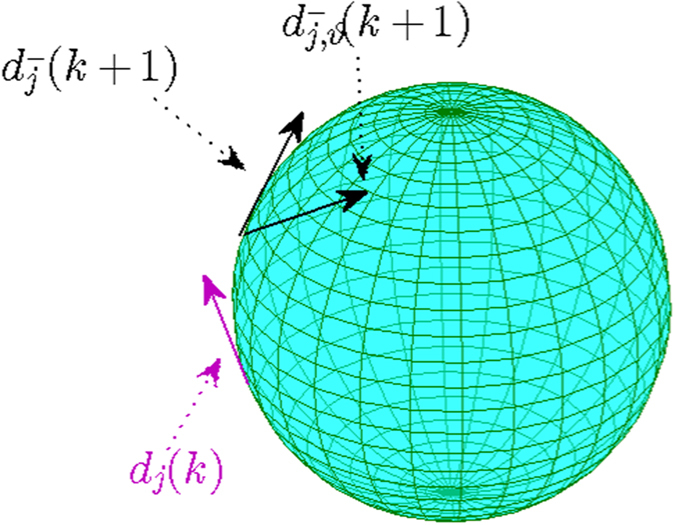
Illustration of directions 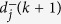 and 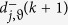 on the tangent plane *T*_*j*_(*k* + 1). The two directions 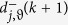, 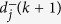 have the noised angle *ϑ*.

Consider the noise in the spherical approaching-directions in calculating the SDA in (12), i.e., a random swing of 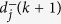 with an angle 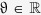 on the *tangent plane T*_*j*_(*k* + 1) for each agent *j* and step *k* (refer to Fig. 1), where *ϑ* represents a white noise with the strength randomly distributed in the interval [−*η*, *η*], *η* ≥ 0 is a constant that models the strength of the noise, note that the unit of *η* is in radians in this paper. Denote 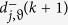 as the noised spherical approaching-direction of 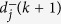, note that 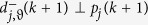, then the SDA rule (12) becomes


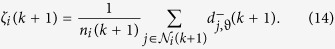


### Calculation of Noise in the SDA

To calculate the noise in the SDA, first, select a value of *ϑ* randomly and uniformly from the interval [−*η*, *η*] for each agent *j* and step *k*, then


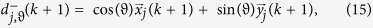


where


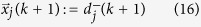


and


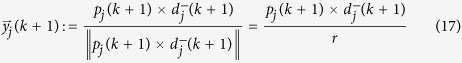


are one pair of the orthogonal X-axis and Y-axis on the tangent plane *T*_*j*_(*k* + 1) at position *p*_*j*_(*k* + 1). Note that 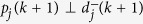. Also note that 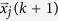

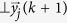
, 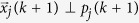, 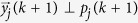, and 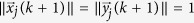. As a result, 
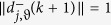
 and 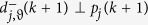.

## Reduction from local sphere to 2D Euclidean space

The topologies of the sphere and the Euclidean space are completely different but have some relations. Consider a very limited local region of the sphere, which seems “flat” and thus approximates to the 2D Euclidean space, then for the agents on a local sphere, some mathematical expressions are similar to the counterparts in the 2D Euclidean space.

As an extreme case, when the radius of the sphere is infinite, the agents evolving on a very local sphere is similar as in the 2D Euclidean space, in this case the vectors in the SCM model reduce to be the corresponding vectors in 2D Euclidean space, then the tangent plane *T*_*j*_(*k* + 1) is always the 2D Euclidean plane, and 
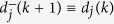
,


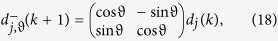


and 
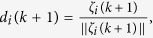
 in which (14) becomes
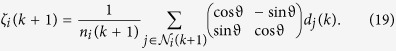
Equation (3) becomes


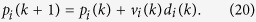


Equation (5) remains. As a result, the model reduces to be the ECM model with the AVM (*α* > 0) in the 2D Euclidean space^27^,^28^.

Its further reduction with *α* = 0 is similar to the famous Vicsek-model (VM)^22^, upon which many variations have been developed. Note that only for the 2D Euclidean case, the direction 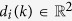 of agent *i* can be expressed as *d*_*i*_(*k*) = [cos *θ*_*i*_(*k*), sin *θ*_*i*_(*k*)]^*T*^ using a parametrized angle, as in many literature, where 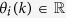 is a scalar angle of agent *i* at step *k*.

## Characterization

There are some characteristics for the SCM that are different from the case for the ECM. For example, the notion of the spherical approaching-direction [refer to Eq. (8)], the new order-parameters, the mean angular-momenta, the manifold-centroid of agents, the principal plane, the principal great-circle, and the scale of spherical distribution of agents, etc. The details are illustrated in the following.

**Figure 2:**
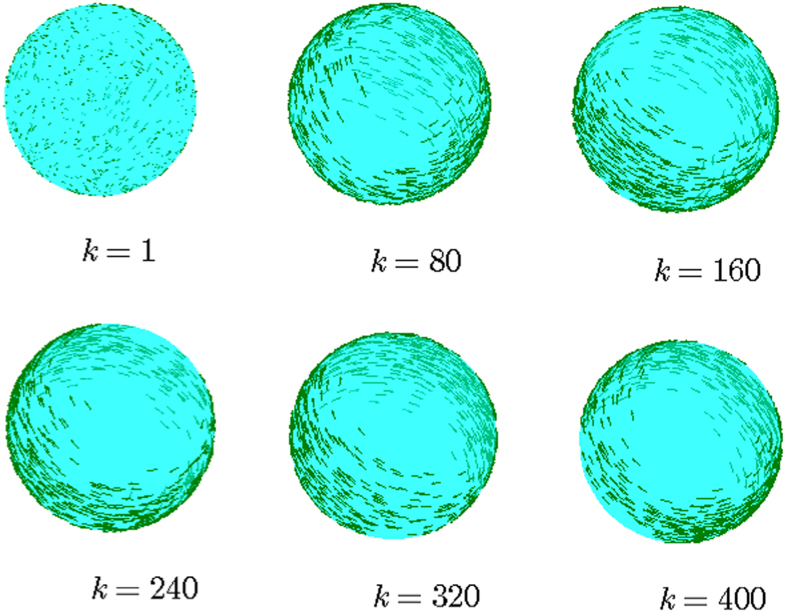
Illustration of the snapshots of one instance of the SCM. *r* = 1, *v*_0_ = 0.02. *n* = 800, *α* = 1, *η* = 0.1, *r*_0_ = 0.4. The agents initially distribute on the whole sphere with uniformly random configurations. The evolutionary characterization is illustrated in Figs 3 and 4.

**Figure 3:**
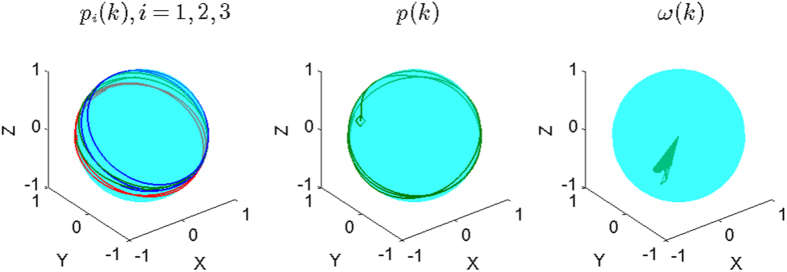
Illustration of the trajectories of three agents among all agents, *p*(*k*) and *ω*(*k*), respectively. The parameters and the initial condition are same as in Fig. 2.

**Figure 4:**
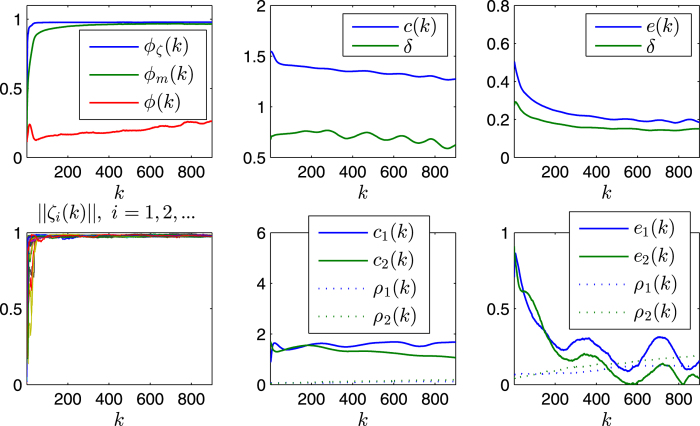
Illustration of some characterization variables. The parameters and the initial condition are same as in Fig. 2. Notation *δ* represents the standard-deviation of the variables *c*_*i*_(*k*) or *e*_*i*_(*k*), *i* = 1, 2,…, *n*, in the corresponding legend, which is the square-root of an unbiased estimator of the variance.

The *usual* order-parameter for the ECM is defined as


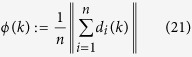


as in most literature, which, however, is less effective (or even improper) for the SCM, especially when the agents are distributed on a large scale of the sphere that is comparable with its radius. For example, this improper aspect can be seen from one instance of the swarming evolution (refer to Fig. 2, with the trajectories of the agents and characteristics illustrated in Figs 3 and 4, respectively), in which the snapshots (Fig. 2) already show the very order of the agents, while the usual order-parameter *ϕ*(*k*) (refer to Fig. 4) is still very low.

One suggested order-parameter for the SCM is the norm of the mean angular-momenta of agents, i.e.,


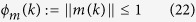


where


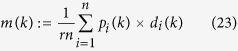


is the mean angular-momenta of agents about the center of the sphere. Certainly, there is no conservation of angular-momentum in self-propelled agents. As an extreme case, *ϕ*_*m*_(*k*) = 1 when all the agents move steady on the same great-circle of the sphere.

Another suggested order-parameter is the average of the *local*-order-parameters of 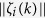 for all the agents:


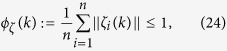


which is effective for the swarm with cohesive motion while less meaningful if the swarm breaks into numerical fragments. Note that


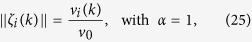


thus


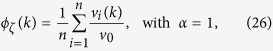


i.e., *ϕ*_*ζ*_(*k*) is just measured by the average adaptive speed of all agents at each step, divided by the maximum speed *v*_0_. The strength of noise in the swarm influences the value of *ϕ*_*ζ*_(*k*), but not the equation (26) of *ϕ*_*ζ*_(*k*).

Define the *manifold-centroid* of agents as:


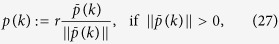


where


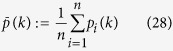


is the average Euclidean position of agents. The swarm has generally no aggregation on a local sphere when 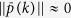.

Denote


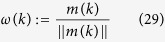


as the *rotation-axis*, denote the *principal plane* 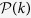 as the plane that is perpendicular to *ω*(*k*) and passes through the center of the sphere, denote the *principal great-circle* as the great-circle of the sphere on 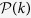.

For the *scale of spherical distribution* of agents, one measure is the mean GCD of agents to *p*(*k*), i.e.,


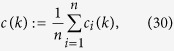


where


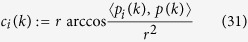


is the GCD between *p*_*i*_(*k*) and *p*(*k*); another is the mean Euclidean-distance of all agents to plane 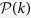, i.e.,


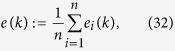


where


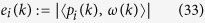


is the Euclidean-distance of agent *i* to plane 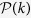. Note that *e*(*k*) < *r*, and *e*(*k*) → 0 means that the agents converge to the principal great-circle of the sphere and rotate about *ω*(*k*).

Denote 
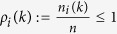
 as the ratio of the number of the agents in the vicinity of agent *i*. Denote 
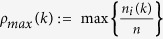
.

## Evolution of SCM with SDA

It is just as expected that, when the distribution-density of the agents is large enough and the noise is weak, the agents will exhibit ordered motion as a whole; otherwise, the agents will break into some ordered fragments at the very beginning of the evolution.

Interestingly, for the evolution of the SCM with the SDA, there are some phenomenons and properties that are distinct from the cases of the ECM.

For example, the fragments will have more opportunity later to collide and merge into a whole swarm, due to the topology of the sphere.

Also, the SCM with the SDA has a distinct shrinking effect, which will make the scale of the swarming agents continuously shrinking during the evolution, even with a large noise (refer to the next section), i.e., the swarm on the sphere has ordered motion with a stronger tolerance of noise.

Figure 2 illustrate one instance of the swarming evolution. During the evolution, the scale of the swarming agents converges, as shown in *c*(*k*) and *e*(*k*), with increasing *ϕ*_*m*_(*k*) and *ϕ*_*ζ*_(*k*) in Fig. 4, which are appropriate order-parameters for the SCM in this case; however, the emergence *cannot* be effectively reflected by the *usual* order-parameter *ϕ*(*k*) that is valid for the ECM [note that *ϕ*(*k*) → 1 only when the agents converge to a very limited local scale on the sphere with ordered motion]. When *r*_0_ is small, the agents will converge approximately to move on the sphere with the trajectories that are approximately parallel to the principal great-circle. As *r*_0_ is large, oscillator trajectories of some agents may appear [e.g., refer to curves of *e*_1_(*k*), *e*_2_(*k*) in Fig. 4, the definition of *e*_*i*_(*k*) is provided in Eq. (33)], except the agents that are very near to plane 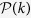.

Generally the agents will never evolve to a consensus velocity (or direction) even without noise (except the trivial case that all the agents always occupy same position at each step), due to the topology of the sphere, this is different from the case of the Euclidean space.

## Statistical Properties

For the statistical properties, consider the SCM at a certain terminal step *k*. Note that the order parameters evolve faster (Fig. 4) and then remain relatively stable, while the scale of the swarm shrinks continuously and slowly. In this paper, *k* = 600 (if without special mention) is set instead of a still larger value, since the properties are relatively stable and the SCM is a computationally incentive simulation.

The state of a swarm at step *k* provides that slice of the evolution. For clarity, denote the values of *ϕ*_*m*_(*k*), *ϕ*_*ζ*_(*k*), *ϕ*(*k*), *e*(*k*), etc., at the terminal step, as *ϕ*_*m*_, *ϕ*_*ζ*_, *ϕ*, *e*, respectively. Each of the order-parameters *ϕ*_*m*_(*k*), *ϕ*_*ζ*_(*k*), *ϕ*(*k*) reflects one aspect of emergence; in many cases, all these order-parameters are required (one serves as a compensation to another) to characterize the emergence.

**Figure 5:**
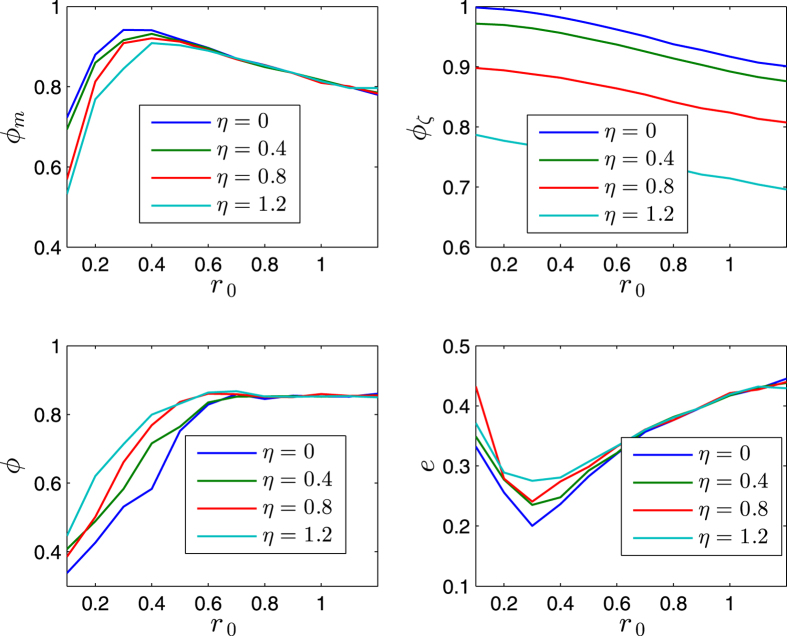
Illustration of the statistical properties as a function of noise *η* ∈ [0, 1.2](*rad*) and *r*_0_ ∈ [0.1, 1.2] × *r*. The x-axis is *r*_0_. *r* = 1. *n* = 600. *v*_0_ = 0.02. The agents initially distribute on the whole sphere with uniformly random configurations. Each date is averaged by 40 runs. *α* = 0. *k* = 600.

**Figure 6:**
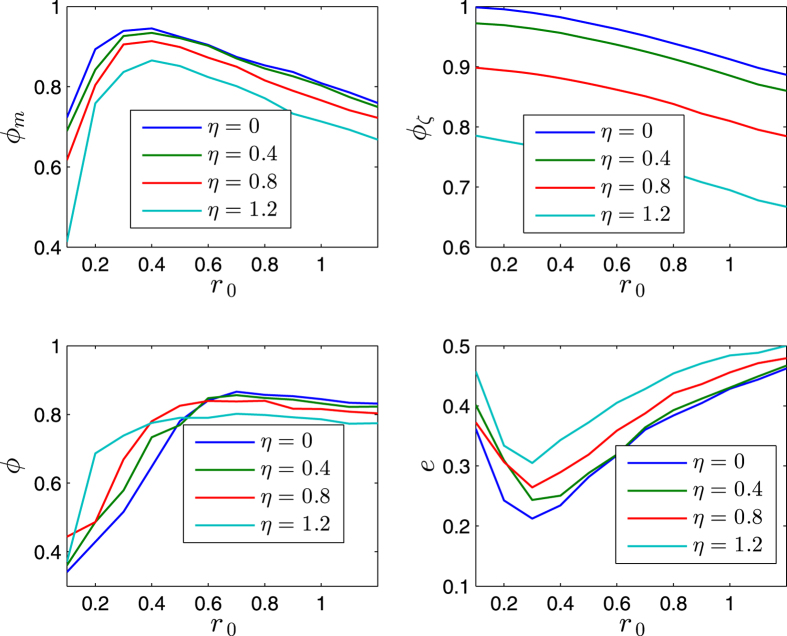
Illustration of the statistical properties as a function of noise *η* ∈ [0, 1.2](*rad*) and *r*_0_ ∈ [0.1, 1.2] × *r*. The x-axis is *r*_0_. *r* = 1. *n* = 600. *v*_0_ = 0.02. The agents initially distribute on the whole sphere with uniformly random configurations. Each date is averaged by 40 runs. *α* = 2. *k* = 600.

Figures 5 and 6 illustrates some statistical properties of the SCM as a function of noise *η* and *r*_0_. For the curves of *ϕ*_*m*_ with different noise, the values of *ϕ*_*m*_ first increase as *r*_0_ increases, and reach the peaks as *r*_0_ is around *r*_*p*_ ≈ 0.4, and then decrease after that. From the simulation results, the transition at the peaks implies the transition of motion patterns that from the pattern of ordered fragments for *r*_0_ < *r*_*p*_ to the pattern of cohesion (i.e., without fragments) for *r*_0_ > *r*_*p*_. The increase of *ϕ*_*m*_ is expected as *r*_0_ increases for *r*_0_ < *r*_*p*_. While for *r*_0_ > *r*_*p*_, the agents move as a whole without fragments; in this case, as *r*_0_ increases, the scale *e* of the swarm increases, thus *ϕ*_*m*_ decreases (due to the topology of the sphere). *ϕ* has a similar but delayed transition. The scale *e* first decreases and then increases. In Figs 5 and 6, *ϕ*_*ζ*_ monotonically decreases as *r*_0_ increases; note that *ζ*_*i*_ measures the local order of agent *i*, thus for a small enough *r*_0_, no matter how many fragments, *ϕ*_*ζ*_ always has a large value; in other words, *ϕ*_*ζ*_ is valid and effective when the agents have no (or less) fragments (*r*_0_ > *r*_*p*_), in this case, as *r*_0_ increases, the scale *e* increases, the local order *ζ*_*i*_ decreases (due to the topology of the sphere), thus *ϕ*_*ζ*_ decreases.

The exponent *α* = 0 makes the values of *ϕ*_*m*_, *ϕ*, and *e* (after the transitions) less influenced by the noise than the case of *α* > 0.

**Figure 7:**
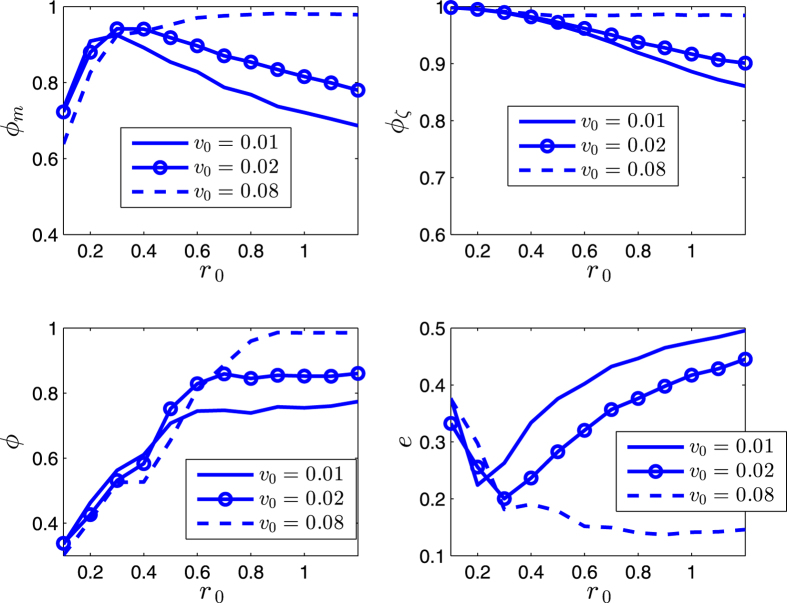
Illustration of the statistical properties of the SCM. *r* = 1. *n* = 600. *α* = 0. The agents initially distribute on the whole sphere with uniformly random configurations. Each date is averaged by 40 runs. *η* = 0. *k* = 600.

**Figure 8:**
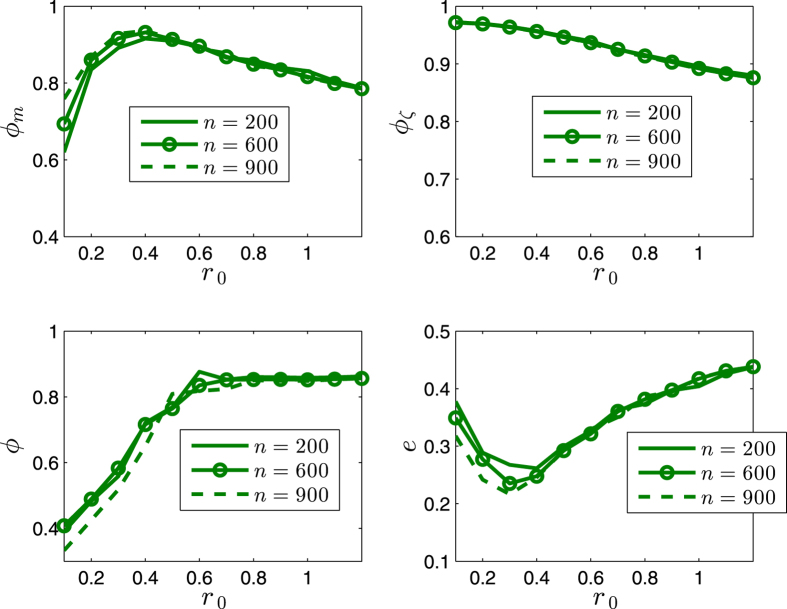
Illustration of the statistical properties of the SCM. *r* = 1. *n* = 600. *α* = 0. The agents initially distribute on the whole sphere with uniformly random configurations. Each date is averaged by 40 runs. *η* = 0.04. *k* = 600.

The effects of *r*_0_ and *v*_0_ are completely opposite, with respect to the influence on the scale of the swarm. For a larger enough *r*_0_, the increase of *r*_0_ tends to increase the scale of the swarm (Figs 5 and 6). While as *v*_0_ increases, the shrinking effect (since all the agents move on the respective great-circles at each step, and no two great-circles are parallel, which tends to make the trajectories of the agents continuously converge, due to the topology of the sphere) of the swarm is strengthened, thus the scale *e* decreases, and the order parameters increase (Figs 7 and 8). As *v*_0_ is larger enough (e.g., Figs 7 and 8 as *v*_0_ = 0.08) that overweights the effect of *r*_0_, the scale *e* decreases monotonically and *ϕ*_*m*_ increases monotonically.

**Figure 9:**
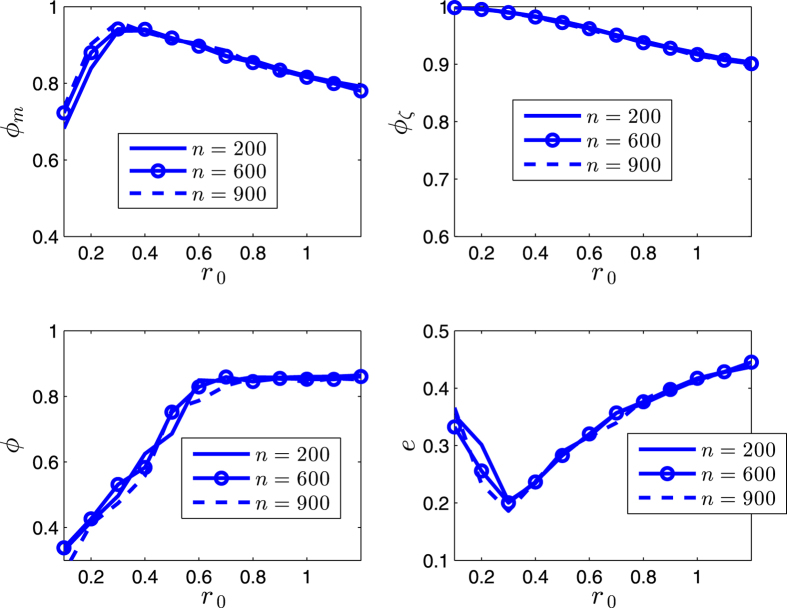
Illustration of the statistical properties of the SCM. *r* = 1. *α* = 0. *v*_0_ = 0.02. The agents initially distribute on the whole sphere with uniformly random configurations. Each date is averaged by 40 runs. *η* = 0. *k* = 600.

**Figure 10:**
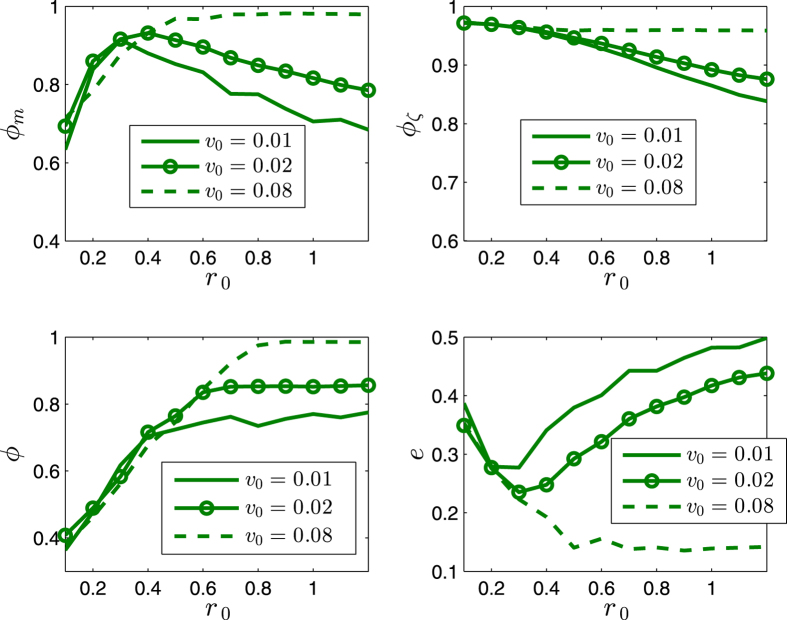
Illustration of the statistical properties of the SCM. *r* = 1. *α* = 0. *v*_0_ = 0.02. The agents initially distribute on the whole sphere with uniformly random configurations. Each date is averaged by 40 runs. *η* = 0.04. *k* = 600.

The order parameters and the scale of the swarm are less influenced by the number *n* of agents (i.e., the different density of the swarming agents) in the swarm as illustrated in Figs 9 and 10.

As the noise increases, the suggested order parameters *ϕ*_*m*_(*k*) and *ϕ*_*ζ*_(*k*) for the SCM decrease, and *e* increases, as expected. However, the usual order parameter *ϕ*(*k*) has a larger value for a larger noise for *r*_0_ < *r*_*p*_ in Figs 5 and 6, this is another perspective to show that the usual order parameter *ϕ*(*k*) is not a good order parameter for the SCM (also refer to the example in the second paragraph of Section V).

## Conclusion

This paper provides a fundamental yet simplest possible and effective framework for the SCM of agents driven by a GCR, which is versatile in the sense that, a variety of concrete cooperative rules of agents can be designed separately and integrated easily into the framework. This paper also designs the SDA, and investigates the unique phenomenons and properties that are specific to the SCM, which unveils an impact of the topology of the sphere on swarming emergence. There are some directions for future investigation. For example, i) the SCM with the SDA has very rich dynamics, with more characteristics that need to be further investigated; ii) other concrete cooperative rules of agents for different motion patterns on a sphere will be considered in a future paper; and iii) the framework has important implications in analyzing the SCM of many types of self-propelled agents on a sphere and even continuum-flows (e.g., by a coarse-grained approximation) for further investigation.

## Additional Information

**How to cite this article**: Li, W. Collective Motion of Swarming Agents Evolving on a Sphere Manifold: A Fundamental Framework and Characterization. *Sci. Rep.* **5**, 13603; doi: 10.1038/srep13603 (2015).
