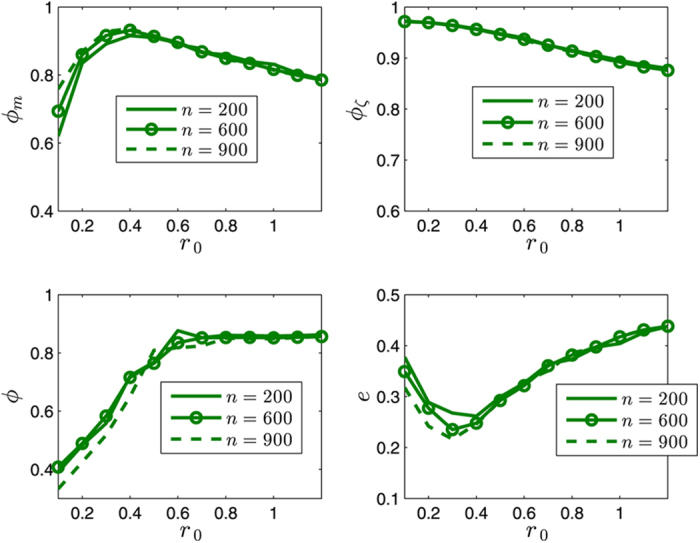# Corrigendum: Collective Motion of Swarming Agents Evolving on a Sphere Manifold: A Fundamental Framework and Characterization

**DOI:** 10.1038/srep15596

**Published:** 2015-10-28

**Authors:** Wei Li

Scientific Reports 5: Article number: 1360310.1038/srep13603; published online: 09 09 2015; updated: 10 28 2015.

This Article contains an error in the order of the Figures. Figures 8 and 10 were published as Figures 10 and 8 respectively. The correct Figures 8 and 10 appear below as Figures 1 and 2. The Figure legends are correct.

## Figures and Tables

**Figure 1:**
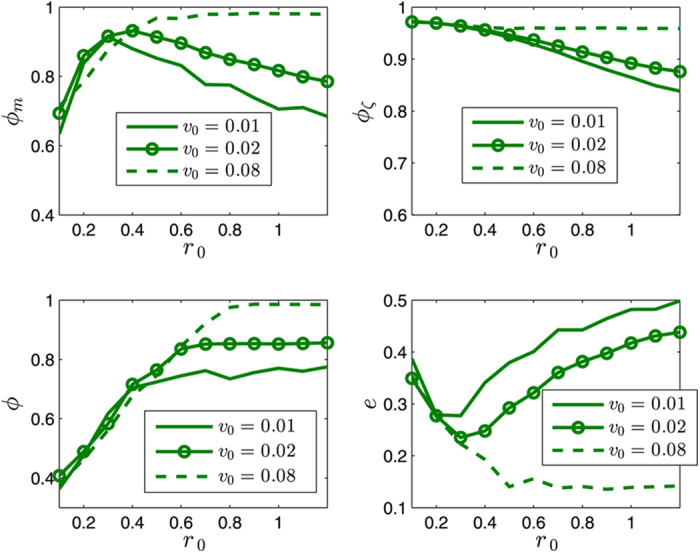


**Figure 2:**